# Life-threatening anaphylactic shock induced by cadonilimab (AK104), a PD-1/CTLA-4 bispecific antibody: a case report

**DOI:** 10.3389/fphar.2026.1834324

**Published:** 2026-08-10

**Authors:** Zhiming Zheng, Jianrong Li, Qiang Huang

**Affiliations:** 1 Xiaolan Clinical Institute of Shantou University Medical College, Zhongshan, China; 2 Department of Pharmacy, Xiaolan People’s Hospital of ZhongShan (The Fifth People’s Hospital of ZhongShan), Zhongshan, China

**Keywords:** anaphylactic shock, bispecific antibody, cadonilimab, cervical cancer, hypersensitivity reaction, immune checkpoint inhibitor, infusion-related reaction

## Abstract

This case report describes a 51-year-old woman with recurrent cervical squamous cell carcinoma who developed acute anaphylactic shock within 5–10 min of initiating intravenous cadonilimab infusion. The physician’s order was placed at 16:04; however, the actual bedside infusion was initiated at approximately 20:00 without routine premedication. Five minutes after infusion start, she presented with pruritus and multiple erythematous maculopapular rashes over the trunk, followed by dyspnea, generalized flushing, profound hypotension (68/40 mmHg), oxygen desaturation (SpO_2_ 82%), and altered consciousness. The infusion was immediately discontinued. Emergency treatment included subcutaneous epinephrine 0.5 mg, intramuscular diphenhydramine 20 mg, intravenous dexamethasone 10 mg, aggressive fluid resuscitation, and supplemental oxygen. Because refractory hypotension persisted, intravenous norepinephrine (4 mg via continuous pump) was initiated and maintained for approximately 72 h. Hemodynamic stability was restored within 45 min. Comprehensive evaluation excluded infection, cardiogenic shock, pulmonary embolism, and cytokine release syndrome. The event fulfilled World Allergy Organization diagnostic criteria for anaphylaxis and was graded as Common Terminology Criteria for Adverse Events (CTCAE) v5.0 Grade 4. Causality assessment using the Naranjo scale yielded a score of 6, indicating a probable adverse drug reaction. The patient had a documented prior hypersensitivity to paclitaxel but no prior exposure to immune checkpoint inhibitors. Rechallenge was not attempted. Cadonilimab may rarely cause rapid-onset, life-threatening anaphylactic shock during the first infusion. Careful monitoring, immediate access to emergency management—including vasopressor support—and heightened awareness of bispecific antibody–associated hypersensitivity are essential.

## 1 Introduction

Immune checkpoint inhibitors have transformed the treatment of advanced malignancies. Cadonilimab is a humanized tetravalent bispecific antibody that simultaneously targets programmed death-1 (PD-1) and cytotoxic T-lymphocyte–associated antigen 4 (CTLA-4), leading to enhanced T-cell activation through dual checkpoint blockade. While immune-related adverse events are well recognized, severe immediate hypersensitivity reactions to cadonilimab remain poorly characterized. A PubMed search using the terms “cadonilimab,” “AK104,” “anaphylaxis,” and “hypersensitivity” identified only a few reports of infusion-related reactions or milder hypersensitivity, with no prior detailed descriptions of life-threatening grade 4 anaphylactic shock during the first infusion. To our knowledge, this is one of the first reported cases of CTCAE grade 4 anaphylactic shock induced by cadonilimab.

## 2 Case presentation

A 51-year-old woman with recurrent metastatic cervical squamous cell carcinoma was admitted on 15 September 2025, for further systemic therapy. She had initially been diagnosed in April 2024 after presenting with abnormal vaginal bleeding. Imaging revealed a cervical mass with bilateral pelvic lymph node and pulmonary metastases. Biopsy confirmed poorly differentiated squamous cell carcinoma, staged as FIGO stage IIIC1. She underwent concurrent chemoradiotherapy (cisplatin) followed by systemic chemotherapy (paclitaxel and cisplatin). However, imaging in early 2025 demonstrated disease progression with persistent pulmonary metastases. Cadonilimab therapy was therefore planned.

She had no prior exposure to immune checkpoint inhibitors but carried a documented history of hypersensitivity to paclitaxel (characterized by skin flushing, diaphoresis, and oral foam during a prior infusion). She had no known food allergies. Her medical history was otherwise unremarkable for asthma, autoimmune disease, or cardiovascular disorders. On admission, her vital signs were stable with blood pressure 98/68 mmHg, heart rate 92 bpm, respiratory rate 20/min, temperature 36.5 °C, and oxygen saturation 98% on room air. Laboratory tests showed hemoglobin 89 g/L, leukocytes 4.31 × 10^9^/L, platelets 236 × 10^9^/L, and normal hepatic, renal, and cardiac biomarkers. Electrocardiography revealed normal sinus rhythm. Chest CT excluded pulmonary embolism or active infection.

On 17 September 2025, a physician’s order for intravenous cadonilimab was placed at 16:04. After completion of pre-infusion assessments and patient preparation, the actual bedside infusion was initiated at approximately 20:00 without routine premedication. Five minutes after the infusion began, the patient abruptly developed pruritus and multiple erythematous maculopapular rashes over the trunk. Typical urticarial wheals were not observed. Within minutes, symptoms progressed to chest tightness, dyspnea, dizziness, and altered consciousness. At 20:10 (approximately 10 min after infusion start), she was diaphoretic with generalized cold sweat and appeared pale. Blood pressure was 113/60 mmHg, heart rate 130 bpm, respiratory rate 26/min, and oxygen saturation 82%. The infusion was immediately discontinued.

At 20:07, diphenhydramine 20 mg intramuscular and dexamethasone 10 mg intravenous had been administered. At 20:10, subcutaneous epinephrine 0.5 mg was given promptly, along with rapid intravenous isotonic saline infusion and oxygen supplementation (6 L/min). Continuous hemodynamic monitoring was established. Despite these measures, blood pressure fell precipitously to 63/40 mmHg at 20:12, with heart rate increasing to 140 bpm and oxygen saturation dropping to 78%. Intravenous norepinephrine bitartrate (4 mg via continuous pump) was therefore initiated as a vasopressor and maintained for hemodynamic support.

By 20:45, the patient’s condition had improved markedly. She was conscious and oriented, with clear vision, no dyspnea, and no pruritus. Blood pressure recovered to 109/53 mmHg, heart rate decreased to 103 bpm, and oxygen saturation rose to 98% with oxygen therapy. The trunk rashes had faded. Serial cardiac biomarkers and electrocardiography remained normal. There was no clinical evidence of pulmonary embolism, myocardial infarction, or cytokine release syndrome. The patient was closely monitored in an intensive care setting for 24 h without recurrence. The event was classified as CTCAE v5.0 Grade 4 infusion-related reaction consistent with anaphylactic shock. Cadonilimab was permanently discontinued. Norepinephrine was gradually weaned and stopped on September 20. She was discharged in stable condition on 22 September 2025 (Table 1).

**TABLE 1:** Minute-by-minute timeline of acute reaction.

Time after infusion	Clock time	Event	Blood pressure	Heart rate	SpO_2_	Intervention
0 min	−20:00	Infusion initiated	95/60	98	98%	—
+5 min	20:05	Pruritus; erythematous maculopapular rashes on trunk	Not recorded	Not recorded	Not recorded	Infusion stopped; IV line changed to normal saline
+7 min	20:07	Chest tightness; dyspnea; dizziness	113/60	120	88%	Diphenhydramine 20 mg IM; dexamethasone 10 mg IV
+10 min	20:10	Shock state; altered consciousness	113/60	128	82%	Epinephrine 0.5 mg SC; oxygen; aggressive IV fluid resuscitation
+12 min	20:12	Refractory hypotension	63/40	105	96%	Norepinephrine 4 mg IV pump initiated
+45 min	20:45	Stabilized	109/53	96	98%	Monitoring; continue norepinephrine

Blood pressure was 113/60 mmHg upon initial assessment at 20:10, but rapidly collapsed to 63/40 mmHg by 20:12 despite immediate resuscitation, reflecting the fulminant nature of the reaction. IM, intramuscular; IV, intravenous; SC, subcutaneous.

## 3 Discussion

Immune checkpoint inhibitors (ICIs) have substantially improved outcomes across multiple malignancies, including recurrent cervical cancer (Postow et al., 2018; Michot et al., 2016). Although generally better tolerated than cytotoxic chemotherapy, ICIs are associated with immune-related adverse events (irAEs) that may affect nearly any organ system (Postow et al., 2018; Michot et al., 2016). Most irAEs are delayed autoimmune phenomena; however, acute infusion-related hypersensitivity reactions, while uncommon, can be rapidly progressive and life-threatening (Chung, 2008; Castells, 2009; Storgard et al., 2024). Reported rates of severe infusion reactions to PD-1 or CTLA-4 inhibitors are typically below 1%–2%, and fatal anaphylaxis remains rare (Postow et al., 2018; Castells, 2008; Yun et al., 2023). As newer immune-modulating agents and bispecific antibodies are introduced into routine practice, continued real-world safety evaluation is essential (Yun et al., 2023).

Cadonilimab (AK104) is a humanized tetravalent bispecific antibody simultaneously targeting PD-1 and CTLA-4, thereby enhancing T-cell activation through dual checkpoint blockade (Zhuang and Liu, 2026). Dual inhibition may provide synergistic antitumor activity but may also amplify immune activation compared with monotherapy (Larkin et al., 2015; Wolchok et al., 2013). Structurally, bispecific antibodies are more complex than conventional IgG monoclonal antibodies, often incorporating modified Fc domains and multivalent antigen-binding regions (Baker et al., 2010). Increased molecular complexity and novel structural configurations have been associated with heightened immunogenic potential in therapeutic proteins (Baker et al., 2010). Although clinical trials of cadonilimab have reported manageable safety profiles (Zhuang and Liu, 2026), rare acute hypersensitivity reactions may not be fully characterized in pre-approval studies, underscoring the importance of post-marketing pharmacovigilance (Yun et al., 2023; Zhuang and Liu, 2026).

Monoclonal antibody–related anaphylaxis can arise through multiple mechanisms. The classical pathway involves IgE-mediated mast cell and basophil degranulation, resulting in rapid hypotension, bronchospasm, mucocutaneous flushing, and gastrointestinal symptoms (Cardona et al., 2020). Alternatively, complement activation–related pseudoallergy (CARPA) may occur independently of IgE through activation of the complement cascade, leading to cardiopulmonary instability (Szebeni, 2014). Direct non–IgE-mediated mast cell activation and cytokine-driven inflammatory responses have also been described in association with biologic therapies (Labella and Castells, 2021). According to the World Allergy Organization criteria, the rapid onset of cardiovascular collapse combined with respiratory compromise strongly supports a diagnosis of anaphylaxis (Cardona et al., 2020). In the present case, profound hypotension, hypoxemia, generalized flushing, and prompt response to epinephrine within minutes of symptom onset are highly consistent with acute anaphylactic shock (Cardona et al., 2020).

An important differential diagnosis is cytokine release syndrome (CRS), which may occur with immune-activating therapies. CRS typically presents with fever, hypotension, and elevated inflammatory markers, often evolving over hours rather than minutes (Lee et al., 2014). In contrast to anaphylaxis, CRS is not primarily mediated by mast cell degranulation and does not usually manifest with prominent urticaria or bronchospasm (Lee et al., 2014). The absence of fever, the extremely rapid clinical deterioration (5–10 min after infusion start), and the immediate response to epinephrine favor anaphylaxis rather than CRS in this patient. Furthermore, baseline cytokine data obtained 1 day prior to infusion (IL-6 21.31 pg/mL, IL-8 69.82 pg/mL, TNF-α 4.85 pg/mL) did not indicate pre-existing hyperinflammation, and post-reaction testing showed no acute CRP surge (0.58 mg/dL vs. 0.69 mg/dL on admission). Although acute-phase ferritin and post-event IL-6 were not tested—an acknowledged limitation—the overall clinical picture is more consistent with anaphylaxis than CRS (Lee et al., 2014).

The management of this case warrants detailed discussion. Current WAO and EAACI guidelines recommend intramuscular (IM) epinephrine as the first-line treatment for anaphylaxis, administered promptly into the anterolateral thigh (Cardona et al., 2020). In this case, the physician’s order for cadonilimab was placed at 16:04, but the actual infusion was not initiated until approximately 20:00. At 20:05 (roughly 5 min after infusion start), the patient developed pruritus and erythematous maculopapular rashes over the trunk, followed by chest tightness and dyspnea. By 20:10 (approximately 10 min after infusion start), she had progressed to profound shock with blood pressure 68/40 mmHg and oxygen saturation 82%. Diphenhydramine 20 mg IM and dexamethasone 10 mg IV were administered at 20:07, followed by subcutaneous (ih) epinephrine 0.5 mg at 20:10. Rapid intravenous fluid resuscitation and high-flow oxygen were provided. Because refractory hypotension persisted (63/40 mmHg at 20:12), intravenous norepinephrine bitartrate (4 mg via continuous pump) was initiated at 20:14 and maintained until September 20 (approximately 72 h). Hemodynamic stability was restored by 20:45 (BP 109/53 mmHg).

While the subcutaneous route of epinephrine deviates from current WAO guidelines recommending IM administration (Cardona et al., 2020), the subsequent need for prolonged norepinephrine infusion reflects the severity and refractory nature of this event. This prolonged hemodynamic collapse—unusual even in severe anaphylaxis—may reflect the unique immunogenic properties of bispecific antibodies or individual patient susceptibility (Baker et al., 2010; Labella and Castells, 2021). We emphasize that infusion facilities must ensure immediate availability of not only IM epinephrine, antihistamines, corticosteroids, and oxygen, but also intravenous vasopressor infusion capability and intensive care monitoring for patients receiving novel bispecific agents (Castells, 2009; Cardona et al., 2020).

From a clinical standpoint, this case emphasizes several practical considerations. Close monitoring is essential during the initial infusion of cadonilimab, particularly within the first 30 min when severe reactions typically manifest (Chung, 2008; Castells, 2009). In this patient, life-threatening anaphylaxis developed within 5–10 min of the actual infusion start, despite the several-hour delay between order placement and bedside administration. According to the Chinese prescribing information for cadonilimab (开坦尼®), premedication (e.g., antihistamines, corticosteroids) is advisable but not mandatory for the first infusion; skin testing is not routinely recommended (Biopharma, 2022). At our institution, premedication is optional unless the patient has a documented history of drug allergy. The occurrence of CTCAE Grade 4 anaphylaxis during the first administration in a patient without known prior sensitization suggests that enhanced protocols—such as universal premedication, prolonged observation, or stepwise infusion rate escalation—should be considered for bispecific immune checkpoint inhibitors (Chung, 2008; Castells, 2009; Biopharma, 2022). According to CTCAE v5.0, Grade 4 infusion-related reactions require urgent intervention and permanent discontinuation of the causative agent (U.S. Department of Health and Huma n Services, 2017). Rechallenge after life-threatening anaphylaxis is generally contraindicated, and desensitization should be considered only in highly selected cases under specialist supervision (Castells, 2009; Labella and Castells, 2021). The patient in this case explicitly refused any future exposure to cadonilimab and opted for alternative systemic therapy after multidisciplinary discussion.

Causality assessment using the Naranjo Adverse Drug Reaction Probability Scale yielded a score of 6 (Probable), itemized as follows: (1) previous reports of this reaction with cadonilimab: No (0); (2) event appeared after drug administration: Yes (+2); (3) reaction improved after drug discontinuation or specific antagonist administration: Yes (+1); (4) rechallenge positive: Not performed (0); (5) alternative causes excluded: No (+2); (6) reaction reappear with placebo: Not applicable (0); (7) toxic blood drug concentration: No (0); (8) dose-response relationship: Not applicable (0); (9) previous similar reaction to the same or similar drug: No (0); (10) objective evidence confirming the adverse reaction: Yes (+1).

This report has several limitations. First, mechanistic biomarkers such as serum tryptase, histamine, or complement levels were not obtained during the acute phase, precluding definitive pathway characterization among IgE-mediated anaphylaxis, CARPA, and non–IgE-mediated mast cell activation (Cardona et al., 2020; Szebeni, 2014; Labella and Castells, 2021). Second, anti-drug antibody testing was unavailable (Baker et al., 2010). Third, the initial administration of subcutaneous rather than intramuscular epinephrine deviates from current WAO guidelines, although the subsequent need for norepinephrine infusion reflects the severity and refractory nature of the event (Cardona et al., 2020). Fourth, acute-phase inflammatory markers (e.g., post-event IL-6, ferritin) were not tested, limiting mechanistic distinction from CRS (Lee et al., 2014). Fifth, the exact infusion rate could only be estimated retrospectively. Nonetheless, the strong temporal relationship, exclusion of alternative etiologies, clinical presentation consistent with established diagnostic criteria, and detailed hemodynamic course collectively support a probable drug-induced anaphylactic shock (Cardona et al., 2020).

As bispecific immune checkpoint inhibitors gain broader clinical application, systematic reporting of rare but severe adverse events is essential to refine their safety profiles (Yun et al., 2023; Zhuang and Liu, 2026). Heightened awareness among oncologists, pharmacists, and infusion teams may facilitate early recognition and timely intervention, thereby mitigating morbidity and mortality associated with catastrophic hypersensitivity reactions (Chung, 2008; Castells, 2009; Cardona et al., 2020).

## Data Availability

The raw data supporting the conclusions of this article will be made available by the authors, without undue reservation.
